# Integrated genomic and metabolomic profiling of ISC1, an emerging *Leishmania donovani* population in the Indian subcontinent

**DOI:** 10.1016/j.meegid.2018.04.021

**Published:** 2018-08

**Authors:** Bart Cuypers, Maya Berg, Hideo Imamura, Franck Dumetz, Géraldine De Muylder, Malgorzata A. Domagalska, Suman Rijal, Narayan Raj Bhattarai, Ilse Maes, Mandy Sanders, James A. Cotton, Pieter Meysman, Kris Laukens, Jean-Claude Dujardin

**Affiliations:** a Department of Biomedical Sciences, Institute of Tropical Medicine, Antwerp, Belgium; b Department of Mathematics and Computer Science, University of Antwerp, Antwerp, Belgium; c BP Koirala Institute of Health Sciences, Dharan, Nepal; d Wellcome Trust Sanger Institute, Hinxton, United Kingdom; e Department of Biomedical Sciences, University of Antwerp, Antwerp, Belgium

**Keywords:** *Leishmania donovani*, Visceral leishmaniasis, Genome diversity, Metabolomics, Genomics, Multi-omics integration, Virulence

## Abstract

*Leishmania donovani* is the responsible agent for visceral leishmaniasis (VL) in the Indian subcontinent (ISC). The disease is lethal without treatment and causes 0.2 to 0.4 million cases each year. Recently, reports of VL in Nepalese hilly districts have increased as well as VL cases caused by *L*. *donovani* from the ISC1 genetic group, a new and emerging genotype. In this study, we perform for the first time an integrated, untargeted genomics and metabolomics approach to characterize ISC1, in comparison with the Core Group (CG), main population that drove the most recent outbreak of VL in the ISC. We show that the ISC1 population is very different from the CG, both at genome and metabolome levels. The genomic differences include SNPs, CNV and small indels in genes coding for known virulence factors, immunogens and surface proteins. Both genomic and metabolic approaches highlighted dissimilarities related to membrane lipids, the nucleotide salvage pathway and the urea cycle in ISC1 versus CG. Many of these pathways and molecules are important for the interaction with the host/extracellular environment. Altogether, our data predict major functional differences in ISC1 versus CG parasites, including virulence. Therefore, particular attention is required to monitor the fate of this emerging ISC1 population in the ISC, especially in a post-VL elimination context.

## Highlights

• ISC1 is an emerging *Leishmania donovani* population in the Nepalese highlands
• The genome and metabolome of ISC1 are distinct from strains from the most recent visceral leishmaniasis outbreak in the Indian sub-continent
• These differences include important host interaction factors
• The emergence of ISC1 should be closely monitored in the Indian subcontinent

## 1 Introduction

Visceral Leishmaniasis (VL) or kala-azar is a neglected tropical disease globally responsible for 0.2 to 0.4 million cases each year (Alvar et al., 2012). The disease is lethal without treatment and caused by protozoan parasites of the *Leishmania donovani* complex. Transmission occurs by sand flies of the *Phlebotomus* genus. In the Indian subcontinent (ISC), VL is of major concern for public health with >200 million people at risk (Ostyn et al., 2011). The recent whole-genome sequencing and phylogenomic analysis of 204 *Leishmania* strains from the ISC (India, Nepal and Bangladesh), revealed that the most recent VL epidemic in this region were essentially driven by one large *L*. *donovani* population that emerged around 1850 and spread across the lowlands in the Ganges plain. Given its recent origin, this population is genetically very homogeneous (Imamura et al., 2016) and was called the Core Group (CG).

Before 2000, sporadic VL cases were reported in the hilly regions of Nepal (1 km above sea level) and were considered the result of travel, rather than a local transmission cycle. However, more recently reports of VL have been increasing in these Nepalese highlands and transmission has been shown to occur locally (Ostyn et al., 2015). Interestingly, some isolates derived from VL patients in this region belong to a genetically distinct population of *L*. *donovani* (called ISC1), which diverged much earlier than 1850 (Imamura et al., 2016). Rai et al. (2017) investigated the geographic spread of different *L*. *donovani* genotypes over a period of 12 years (2002–2014) in Nepal and showed that the frequency of ISC1 was increasing in the country and that this emerging genotype was also present in the lowlands. Our earlier study showed that out of all sampled ISC1 isolates, 75% of them came from patients relapsing to miltefosine treatment, vs 50% for the CG strains (Imamura et al., 2016). We have also demonstrated experimentally that ISC1 parasites need more time and molecular changes to develop antimonial (Sb) resistance *in vitro* than CG strains, which led to the hypothesis of Sb-resistance pre-adaptation of the CG, but not of ICS1 (Dumetz et al., 2018).

Since 2005, the kala-azar elimination programme (KAEP) has been running in India, Nepal and Bangladesh, based on vector control and detection/treatment of VL cases. In such a programme, it is essential to track parasites and in particular to characterize emerging populations like ISC1 or the parasites recently reported in Sri Lanka (Zhang et al., 2014), as these could react differently to control tools like diagnostics, drugs or vaccines. In this context, we report here the results of a deep molecular comparison between ISC1 and CG parasites. In the present study, we focused on parallel untargeted genomics and metabolomics, hereby undertaking the first *Leishmania* diversity study integrating these two ‘omics layers.

## 2 Methods

### 2.1 Parasites

Nine L. *donovani* strains cloned from seven Nepalese and two Indian clinical isolates were analyzed in this study (Supplementary Table S1.1). Among these are five ISC1 strains (BPK026/0, BPK604/0, BPK612/0, BPK519/12, BPK406/6) and two Nepalese (BPK275/0, BPK282/0) as well as two Indian (BHU568/0, BHU573/0) strains from the CG. Promastigotes of all strains were grown in modified Eagle's medium (Invitrogen) supplemented with 20% (*v*/v) heat inactivated fetal calf serum (PAA Laboratories GmbH, Linz, Austria) pH 7.5 at 26 °C. At each passage, all parasite lines were inoculated to a starting concentration of 5∙10^5^ parasites/mL and growth was followed for two passages enabling synchronized sampling of the different cultures and minimizing the effect of differences in growth rate between strains. For each strain, four growth replicates were cultured for metabolomics analysis and one for genome sequencing. Metabolites were extracted in stationary growth phase according to t'Kindt et al. (2010) and DNA with the QIAamp DNA Blood Mini Kit (Qiagen).

### 2.2 Genomics

#### 2.2.1 Library preparation and sequencing

Library preparation and sequencing for BPK026, BPK604, BPK612, BPK512, BPK406 was performed at the Wellcome Trust Sanger Institute (Hinxton, United Kingdom). Genomic DNA was sheared into 400–600-base pair fragments by focused ultrasonication (Covaris Adaptive Focused Acoustics technology (AFA Inc., Woburn, USA)) and standard Illumina libraries were prepared. 125 base pair paired end reads were generated on the HiSeq 2000 v4 according to the manufacturer's standard sequencing protocol (Bronner et al., 2014). Libraries of BPK275, BPK282, BHU568 and BHU573 were prepared and sequenced at the Beijing Genomics Institute (BGI). Briefly, mechanical shearing with the Covaris (Illumina) was used to create 250-350 bp fragments, after which the fragment ends were repaired and 3′ A-tailed. Adapters were subsequently ligated and the target fragments were recovered from a 2% agarose gel after electrophoresis. Finally, the libraries were PCR amplified and 2 × 151 BP sequenced on an Hiseq 4000. All sequencing data of this study was submitted to the Sequence Read Archive (SRA) under accession number SRP125482. Individual accession numbers for each sample are available in Supplementary Table S2.

#### 2.2.2 Data analysis

Genomic analysis was performed as described in Dumetz et al. (2017). Briefly, reads were mapped to the L. *donovani* LdBPKv2 reference genome (9) using Smalt v7.4 (www.sanger.ac.uk/resources/software/smalt/) and the options exhaustive searching (−x), read identity threshold of 80% (−y 0.8) and random mapping of multiple hits reads. Duplicate reads were marked with Picard v1.85 and SNPs and small indels were called using the GATK haplotypecaller v3.4 (McKenna et al., 2010). Low quality SNPs were marked using GATK Variant Filtration with paramers: “QD < 2.0 || MQ < 40 || FS > 60.0 || ReadPosRankSum < -8.0” and removed from further analysis. The same tool was used for filtering indels, but with parameters “QD < 2.0 || FS > 200.0 || ReadPosRankSum < -20.0 || SOR > 200”. Variants were annotated with SnpEff v4.1 (Cingolani et al., 2012). SNPs and small indels were considered significantly different between CG and ISC1, when the allele shift difference was at least 0.25 (Tihon et al., 2017) and Mann–Whitney *U* test *p*-value <0.05. Allele shifts larger than 0.80 were considered homozygous variants (Tihon et al., 2017). Local copy number variation and chromosomal somy were determined according to Downing et al. (2011). For somy estimation, initially, the median read depth of each chromosome was calculated (d_i_). All positions with a read depth >1 standard deviation away from this initial median were then removed and d_i_ was recalculated. This approach removed depth outliers by assembly errors, local CNVs or spurious high coverage regions impacting the final median. Subsequently, the median depth of the 36 chromosomes d_m_ was calculated and the somy (*s-value*) of each chromosome was obtained with the following formula *s* = 2*d_i_/d_m_. Haploid gene copy number was defined to be the median depth of a gene divided by the median depth of the chromosome on which it is located. We used two criteria to evaluate whether a gene or chromosome copy number difference between the CG and ISC1 strains was biologically meaningful and statistically significant. The first requirement was that the absolute difference in gene/chromosome copy number between CG and ISC1 should be at least 0.5 (Dumetz et al., 2017). Secondly, the FDR adjusted *p*-value (Student *t-*test + Benjamini Hochberg correction) had to be lower than 0.05.

### 2.3 Metabolomics

#### 2.3.1 Liquid chromatography and mass spectrometry

Samples were analyzed with LC-MS using an Orbitrap Exactive mass spectrometer (Thermo Fisher) coupled to a 2.1 mm ZIC-HILIC column at Glasgow Polyomics (University of Glasgow, Scotland) (Sequant), exactly as previously described (Berg et al., 2015). Both negative and positive ion modes were run in parallel with rapid polarity switching. Several quality control samples were run at the start, middle and end of the LC-MS run to aid accurate metabolite identification and verify LC-MS stability (Berg et al., 2013a). These included: 1) Authentic standard mixes containing in total 217 metabolites (50–400 Da) representing a wide array of metabolic classes and pathways. 2) An amino acid standard mix (Sigma Product No. A9906). 3) Serial dilutions (Undiluted, 1/2, 1/4, 1/8 and 1/16) of a pooled sample of all extracts to filter out spurious signals that do not follow the dilution trend (Jankevics et al., 2012).

#### 2.3.2 Data preprocessing

Data analysis was performed using the XCMS 1.42.0 (Smith et al., 2006) and mzMatch 1.0.1 (Scheltema et al., 2011) packages in R as described previously (Berg et al., 2013b). In summary, mzXML files were first subjected to retention time alignment using ObiWarp (Prince and Marcotte, 2006) and peak detection with the centWave algorithm (Tautenhahn et al., 2008). Corresponding peaks from the 4 biological replicates were subsequently combined and filtered on a maximum allowed reproducibility standard deviation (RSD) of 0.5. Peaksets from all samples were then combined and filtered further, requiring for each peak a minimal scaled CoDA-DW quality value of 0.8 (mzMatch noisefilter), detection in at least 3 out of 4 replicates and a minimal peak intensity of 3000. The gapfiller tool was used to fetch peaks from the mzXML files that were missing in one or more samples and derivative peaks were annotated (Scheltema et al., 2009). The peaks were then putatively identified allowing 2 ppm of mass deviation using sequentially the LeishCyc database (Doyle et al., 2009), LipidMAPS (Fahy et al., 2007), KEGG (Kanehisa et al., 2017), the peptide database included in mzMatch and the Human Metabolome Database (Wishart et al., 2007). Each time, only unmatched peaks were aligned against the next database, reducing the number of potential identifications to the most likely candidates (Scalbert et al., 2009). Peaks of which the intensity did not follow the dilution pattern in the pooled sample dilution series (Pearson correlation >0.8 or *p-*value >.05) were removed from the analysis with the mzMatch Dilution Trend Filter tool (Jankevics et al., 2012). The remaining peaks were normalized for total ion count and visually verified before exporting them to a csv file containing all peak heights, retention times and relevant database annotations. The full analysis script and detailed parameter settings are available in Supplementary Table S5. The csv files from the positive and negative ionization mode were then merged. When metabolites were identified in both modes, only the results from the most intense peak were kept.

#### 2.3.3 Data exploration and statistics

For the initial data exploration, principal component analysis (PCA) was performed in R directly on the metabolite intensity values of each sample. Subsequently, the relative expression between the CG and ISC1 strains was calculated for each individual metabolite (Jara et al., 2017). Briefly, first the average metabolite intensity was calculated per strain. Then, the obtained values were again averaged over the ISC1 strains and over the CG strains. Finally, these strain averages were converted into base-2 logarithm ratios between the two populations, henceforth called the ‘Log2 Fold Change’ (Log2FC). A two-sample *t*-test assuming unequal variance was applied to the strain averages to calculate whether or not this Log2FC was significant (*p* < 0.05).

To investigate the overall correlation between genome and metabolome, we calculated three types of pairwise distances between all strains: 1) The SNP distance (S-distance) was defined as the total number of SNP allele changes between a strain pair. In this calculation, a heterozygous SNP was counted as a distance of 1, while a homozygous SNP was counted as a distance of 2. 2) The gene dosage distance (GD-distance) was determined by taking the Euclidean distance between two strains based on all absolute gene copy differences; as such, it combined the effect of CNV and aneuploidy. 3) The metabolic distance (M-distance) was defined as the Euclidean distance based on all metabolite intensities, however, since these intensities have a large, metabolite-specific dynamic range, they were first transformed into *Z*-scores. Analysis of the correlation between the respective distances was performed with a non-parametric Mantel test (Mantel, 1967) with 999 permutations, using the QIIME2 package in Python. Contrary to the classical correlation test, the Mantel test deals with the problem that distances in a distance matrix are not independent from one other. R^2^ values were determined by linear regression analysis with the lm function in R.

## 3 Results

### 3.1 Genome sequence diversity

**Fig. 1:**
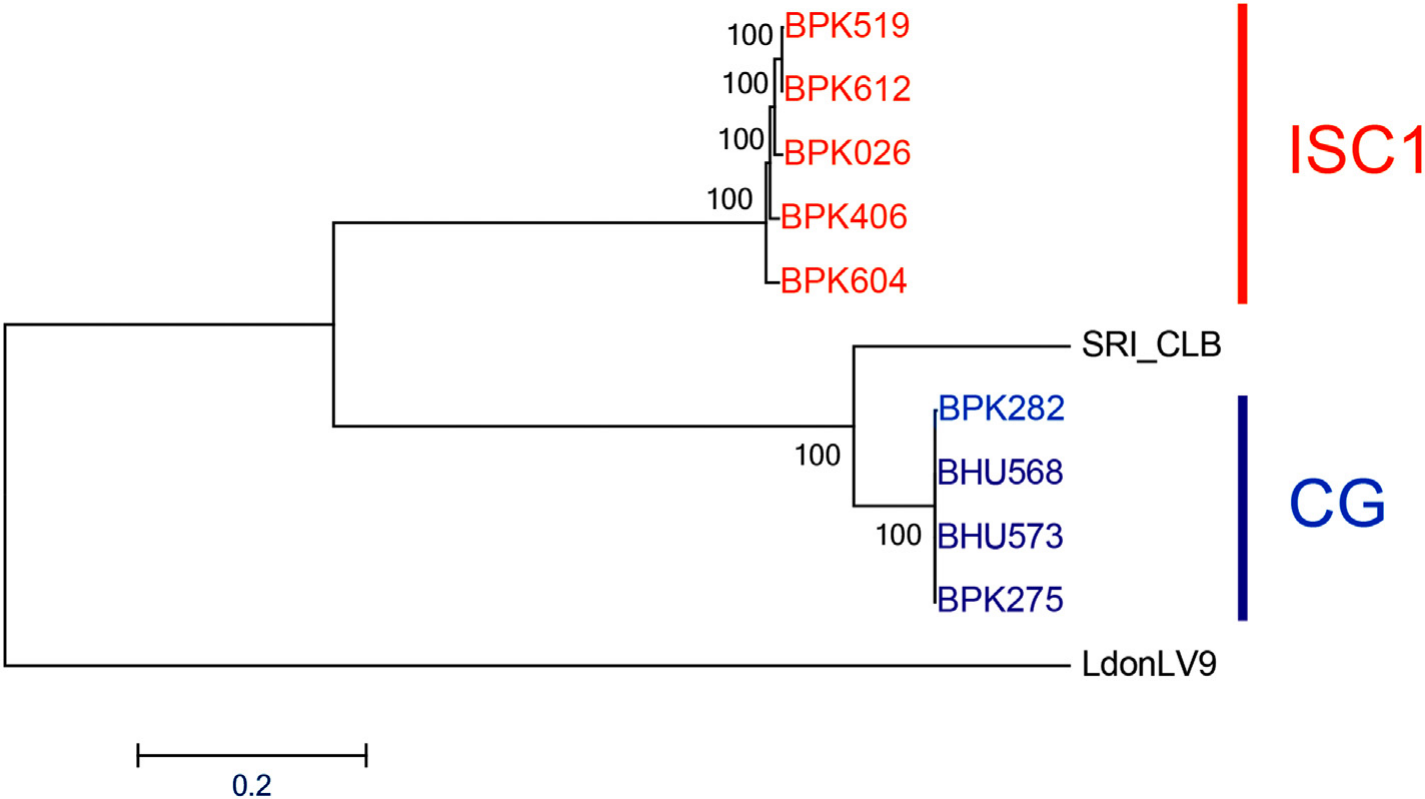
Neighbour joining tree based on whole genome SNP loci. Bootstrap support was calculated on 1000 bootstrap replicates. LdonLV9 = East African L. *donovani* LV9, SRI_CLB = Sri Lankan L. *donovani* SRI_CLB.

Promastigotes of five ISC1 (BPK026/0, BPK604/0, BPK612/0, BPK519/12, BPK406/6) and four CG strains (BPK275/0, BPK282/0, BHU568/0, BHU573/0) were sequenced with an average read depth of at least 38.5× and 97.1% of the genome was covered at least 20×. Detailed mapping to BPK282v2 (Dumetz et al., 2017) and coverage statistics can be found in Supplementary Table S2. Using new SNP data of our strains and published ones of East African *L*. *donovani* LV9 and Sri Lankan *L*. *donovani* SRI-CLB, we performed a phylogenomic analysis (Fig. 1). In correspondence with Imamura et al. (2016) (Imamura et al., 2016), this showed that (i) ISC1 parasites cluster at a larger distance from CG than the Sri Lanka strain SRI-CLB and (ii) in an intermediate position with respect to East African *L*. *donovani* LV9.

In total, we detected 53,101 SNPs between the CG and ISC1 clusters (allele shift >0.25 & *pval* < 0.05) of which 51,668 were homozygous (allele shift >0.8) and of which 19,776 were located in protein coding regions (Supplementary Table S3). 10,550 of these SNPs caused non-synonymous codon changes and 24 were flagged by SnpEff for having a probable high impact on the final protein: fifteen premature stop codons were gained, seven stop codons were lost, and two start codons were gained in ISC1 strains compared to CG ones (Supplementary Table S1.2). Fourteen of these high impact variants concerned hypothetical proteins of which the function and impact is unclear. However, of the remaining ten genes (with homozygous SNPs), three play key roles in *Leishmania* virulence (autophagy protein Atg8, trypanothione synthetase and amastin-like surfaceprotein) and two are involved in phosphatidylinositol metabolism (CDP-diacylglycerol-inositol 3-phosphatidyl transferase and phosphatidylinositol 3- and 4-kinase). While a high impact is predicted by SnpEff, these genes could certainly still be functional, as for many of them (including trypanothione synthase, autophagy protein Atg8 and the amastin-like surface protein) the SNP was found towards the end of the gene.

Also at the indel level major differences were observed between ISC1 and CG with a total of 6435 heterozygous and 5780 homozygous indels (allele shift >0.25 and  > 0.80 respectively, *p*-value < 0.05, Supplementary Table S4). 442 of these indels were located in coding regions and 83 concerned frameshift mutations, which are often highly disruptive. A large proportion (58%) of the frameshift indels were located in hypothetical proteins, while the 33 functionally annotated indels (Supplementary Table S1.3) concerned several important biological functions, including, but not limited to, transmembrane transport (Imamura et al., 2016), translation (Ostyn et al., 2011), nucleic acid binding (Imamura et al., 2016) and nucleotide salvage (Ostyn et al., 2015). 21 of these annotated indels were homozygous mutations. Remarkably, two genes encoding for (i) an AAAfamily ATPAse and (ii) a dihydrouridine synthase domain protein respectively each had two indels. In addition, phosphatidylinositol metabolism was highlighted in both SNP and INDEL analysis: Phosphatidylinositol-specific phospholipase C,X/Y/C2 domain containing protein (indel), CDP-diacylglycerol-inositol 3-phosphatidyl transferase (SNP) and phosphatidylinositol 3-and 4-kinase (SNP).

### 3.2 Genome structure diversity

**Fig. 2:**
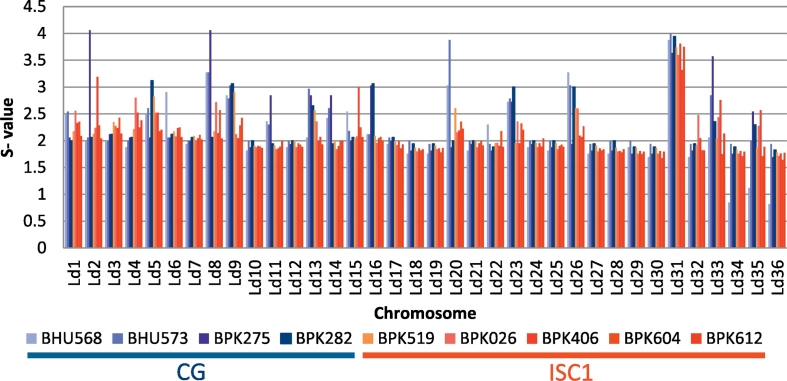
Somy levels in ISC1 and Core Group strains (CG). ISC1 strains are depicted in shades of red and yellow, while CG strains in shades of blue. (For interpretation of the references to colour in this figure legend, the reader is referred to the web version of this article.)

The genome structure was first investigated at chromosome level (Fig. 2). Chromosomal somy estimates do not always correspond to integer values as these represent the average of a population of cells that do not necessarily show identical karyotypes (mosaicism). Many somy differences were found between chromosomes of different strains, but only two of these were consistent between ISC1 and CG populations (*p*-value < 0.05 & |somy difference| > 0.5). The ISC1 strains had 0.8 copies less of chromosome 8 and 0.7 copies less of chromosome 23.

Secondly, we analyzed local gene copy number variations (CNVs) between ISC1 and CG isolates. These differences are the result of gene amplification or deletion events since the most recent common ancestor of ISC1 and CG. For every gene and sample, copy numbers per haploid genome are available in Supplementary Table S4. By expressing copy number differences per haploid genome, we can describe these changes independently of the aneuploidy phenomenon. As *Leishmania* is diploid for most chromosomes, we used 0.5 copies/haploid genome as a cut-off, which corresponds with a single copy for diploid chromosomes. In total we found 62 loci containing genes with CNVs: for 29 of these loci, genes were more abundant in ISC1, while for the 33 remaining loci, genes were more abundant in CG. 29 loci concerned single gene duplications or deletions (Supplementary Table S1.4). The remaining 33 loci contained multiple genes showing CNVs: (i) 22 contained tandem arrays of the same gene family, expanding or contracting (Supplementary Table S1.5); (ii) 11 loci contained sets of genes of a different family, all amplified/deleted together (Supplementary Table S1.6).

Duplication/deletions of single copy genes concerned a wide array of biological functions (Supplementary Table S1.4). Noteworthy, 43% of the genes with known functions were coding for surface proteins, virulence factors or immunogens, including GP63, amastins, 60S acidic ribosomal protein P2 and surface antigen 2. With respect to tandem arrays of the same gene family (Supplementary Table S1.5), a large proportion (37%) of the genes with known functions were also coding for surface proteins, virulence factors or immunogens. More specifically, we found CNVs of genes coding for amastins, proteophophoglycans, HSP70, HSP83, GP63, p1/s1 nucleases, lorien proteins, cysteine peptidase B and surface antigen 2 proteins. Interestingly, some of these CNVs were linked to the observations at sequence diversity level. Indeed, frameshift indels were found in both LdBPK_300020700 coding for a p1/s1 nuclease, and in LdBPK_120014400 coding for a surface antigen protein 2. For both genes the adjacent loci contained genes of the same gene family involved in a CNV. Other families that were targeted by both sequence and structure variants were amastins and autophagy protein Atg8. Finally, amplifications concerning groups of genes with different functions (Supplementary Table S1.6) are extensively reported to occur as circular episomes (Grondin et al., 1993; Mukherjee et al., 2007; Chiquero et al., 1994), but also as intra-chromosomal amplicons (Imamura et al., 2016; Olmo et al., 1995). For instance, the H- and M-loci are amplified in CG strains but have only a single copy per haploid genome in ISC1. Noteworthy, the H-locus contains among others the gene encoding for arginine-succinate synthase (ASS, see metabolomics section). Reciprocally, the R-locus (Ubeda et al., 2014) has a single copy per haploid genome in CG strains, but has 2.15 copies per haploid genome in ISC1 strains. Both the H- and R- locus have been associated with drug resistance (Grondin et al., 1993; Kundig et al., 1999). Five of the other amplified gene groups are associated with virulence, including proteophosphoglycan, amastins, surface antigen protein 2 and acid phosphatases. While all three described types of CNVs concerned a large proportion of genes coding for surface proteins, virulence factors and immunogens there was no clear directionality to these CNVs since we observed both deletions (60%) and duplications (40%) in ISC1 versus CG.

### 3.3 Metabolomics

**Fig. 3:**
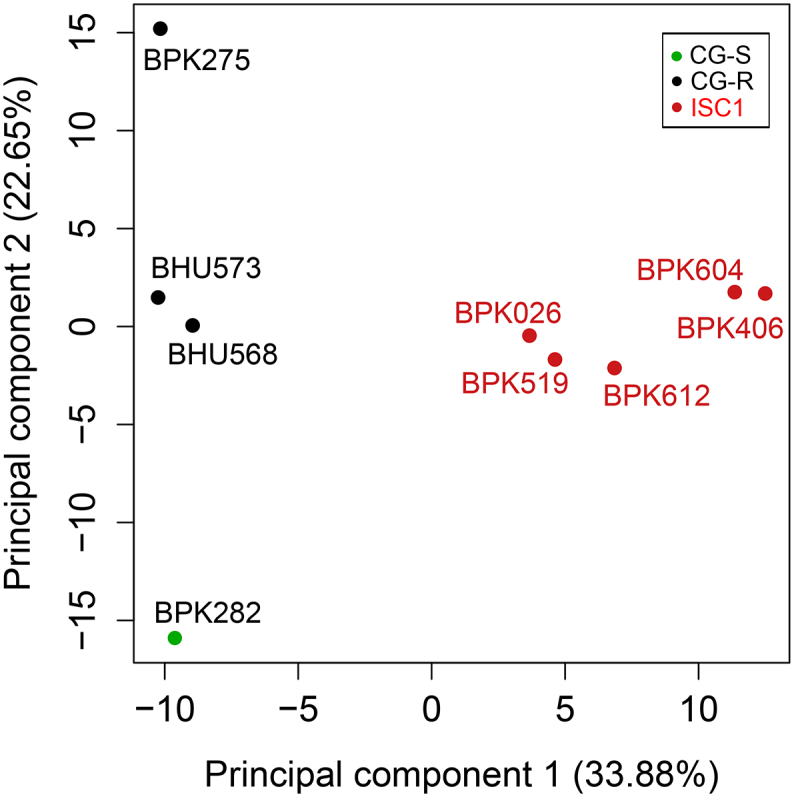
Principal component analysis of the metabolic profiles of the 5 *Leishmania donovani* ISC1 isolates and 4 CG strains in this study. This analysis was based on the quantitative measurements of all 290 putatively identified metabolites. In this PCA plot, average values are shown for each strain based on four biological replicates. For each principal component, the proportion of variation of the total dataset it explains is mentioned.

In total, 290 metabolites were identified with a mass accuracy <2 ppm, covering a broad diversity of metabolic pathways and representing the following classes (in order of quantitative representation): glycerophospholipids (GPLs), amino acids and derivatives, fatty acyls and carbohydrates, nucleobases and nucleosides, sphingolipids, steroids and derivatives (Supplementary Table S5 Supplementary Table S1.4). As a first data exploration of the variation in our dataset, we performed Principal Component Analysis (PCA, Fig. 3). The first principal component explained 34% of the total variation in our metabolomics experiment and clearly separated the CG (BPK275, BHU573, BHU568 and BPK282) from ISC1 strains (BPK026, BPK406, BPK519, BPK604, BPK612). The second principal component explained 23% of the variation, which separated CG strains in 3 groups: BPK275 (Nepal, SSG-resistant), BHU573-BHU568 (India, SSG-resistant) and BPK282 (Nepal, SSG-sensitive).

**Fig. 4:**
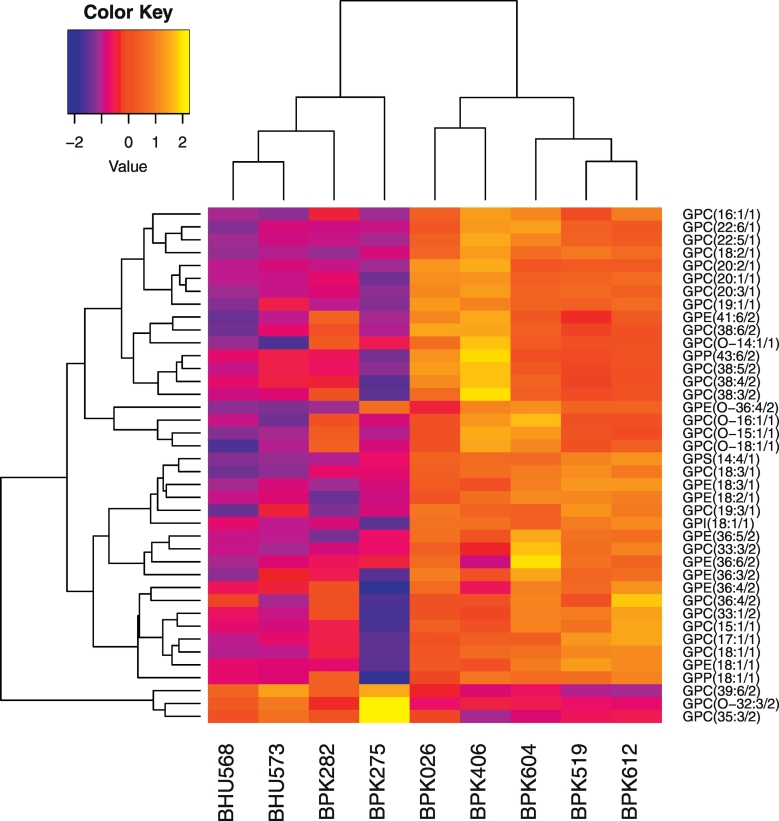
The 40 differentially expressed glycerophospholipids (|Log_2_FC| > 1 & *p* < 0.05) between ISC1 and CG parasites.

33 metabolites were more abundant in ISC1 strains (Log_2_FC > 1, *p*-value < 0.05), while 12 were more abundant in CG strains (Log_2_FC > 1, *p*-value < 0.05). Interestingly, 19 of these metabolites were glycerophospholipids (GPLs), providing evidence for major differences in lipid metabolism between CG and ISC1 (Fig. 4). In addition, ISC1 parasites contained higher metabolite levels of phosphorylethanolamine, glycerylphosphorylethanolamine, and glycerophosphoinositol pointing further towards lipid changes between both groups. Secondly, changes were observed in the urea cycle in ISC1 vs CG. ISC1 had a 2.1 fold higher amounts of citrulline and 3.6 fold lower amounts of argininosuccinate. The reaction that involves these metabolites, namely citrulline + aspartate + ATP - > argininosuccinate + AMP + PPi, is catalyzed by argininosuccinate synthase (ASS). These metabolite differences therefore indicate a lower ASS activity in ISC1, which is in correspondence with the lower copy number of the respective gene in ISC1 (as described in the genome structure diversity section). Interestingly, the metabolite levels also indicate a difference within the nucleotide salvage pathway between both groups, as we found increased levels of guanine, uracil, thymine, AMP in ISC1 vs CG. Finally, concentrations of basic amino acids lysine (up) and histidine (down) were altered in ISC1 vs CG.

We have previously shown that significant metabolic variation is present between SSG-resistant strains (BPK275, BHU573 and BHU568: CG-R) and SSG sensitive strains (BPK282: CG-S) (Berg et al., 2013b). Considering these metabolic differences, we also made individual comparisons of ISC1 vs CG-R, ISC1 vs CG-S and CG-S vs CG-R. As expected, this confirmed substantial differences in the metabolome between CG-S and CG-R, with 52 metabolites showing a significant (Log_2_FC > 1, *p*-value < 0.05) difference in concentration in only one of both groups. Consequently, when comparing CG-R and CG-S to ISC1 individually, we found many metabolites only differential in one of both comparisons (62 and 51 for CG-R and CG-S respectively), that were not observed in the original ISC1 vs CG in comparison. However, when looking in detail to the functions of these newly identified differential metabolites, these related towards the same functional classes of metabolites as those found when comparing ISC1 to all CG strains (Supplementary Table S1.7). This shows that although significant metabolome variation within the CG is present, there are clear and consistent differences with ISC1 on the level of GPLs, salvage pathway and urea cycle.

Finally, we compared the metabolomic and genomic polymorphism to quantify the relationship between both data sets and assess if/how metabolomic variation could be explained by genomic variation. Therefore, we computed distances between each pair of isolates, based on M-distance, S-distance and GD-distance, as explained in the Methods section. Unfortunately, for the S-distance the intra group variance was far smaller than the inter group variance, which biases the results. Therefore we did not analyse S-distance any further. However, this problem was absent for GD-distance and a Mantel test established a significant positive correlation between M-distance and GD-distance (*p*-value = 0.005, Spearman rho = 0.64). This means that GD-distance explains a significant proportion of the metabolic distance, but the low spearman rho indicates that much other variation is present as well.

## 4 Discussion

In this work we have performed a genomic and metabolic profiling study of strains representing ISC1, a new *Leishmania donovani* population that is currently emerging in Nepal. We have demonstrated that these parasites are genomically and metabolically very distinct from the main population (CG) of *L. donovani,* which was the driver of the most recent VL epidemic in the Indian subcontinent (Imamura et al., 2016).

From a genomic point of view, the differences between ISC1 and CG strongly contrast with the homogeneity of the latter group and explained by its young evolutionary history (Imamura et al., 2016). First, we report a large number of SNPs and indels, many of them with a strong impact (stop codons gained/lost and frameshifts) on several important biological functions. Secondly, we also encountered differences in the karyotypes and more particularly two chromosomes (chromosome 8 and chromosome 23) that showed a lower somy in ISC1. Somy variation is an adaptive strategy well known in *Leishmania*, for instance in response to experimental drug resistance selection (Prieto Barja et al., 2017), but its interpretation in clinical isolates needs to be done very carefully. Indeed, we showed that aneuploidy is strongly dependent on the environment and more abundant in promastigotes *in vitro* than in amastigotes within the vertebrate host (Dumetz et al., 2017). Strictly speaking, we can thus only conclude that cultivated promastigotes of ISC1 show significant karyotypic differences to those of CG. Further extrapolation of these karyotype differences to the situation *in vivo* should be tested by direct sequencing of clinical samples or in experimental animal infections. A third major class of genomic changes here observed concerned local copy number variations (CNVs). In contrast to aneuploidy, these were shown not to be affected during the life cycle of the parasite (Dumetz et al., 2017), and CNVs have been shown to be excellent markers for studies on *Leishmania* evolution (Dujardin, 2009), both in experimental (for instance, amplification of specific loci during drug resistance selection (Ubeda et al., 2014; Leprohon et al., 2009)) or in natural settings (for instance, decrease in copy number of gp63, mini-exon and rDNA genes in *Leishmania peruviana* (Victoir et al., 2009; Kebede et al., 1999; Inga et al., 1998)). In the absence of gene expression regulation at initiation (Günzl, 2012), amplification/deletion of specific genes is a genomic solution to modulate the level of transcripts and corresponding products. As such, these CNVs may have an important functional significance. In this context, it is striking to observe that many of the CNVs concerned genes that code for surface proteins, virulence factors or immunogens: most notably, lower copy numbers of amastins, surface antigens and GP63 genes were encountered in ISC1. Interestingly, many *Leishmania* virulence genes were the target of highly impactful non-synonymous SNPs (removed/introduced stop codons), such as autophagy protein Atg8, trypanothione synthetase and amastin-like surface proteins. Although it is impossible to predict now the exact consequences of these SNPs, they are likely to have a high impact at the protein level by extending/shortening the protein. Altogether, genomic differences between ISC1 and CG suggest a potential for differences in virulence, which should be explored by further work. A first experimental verification of this hypothesis was made the context of antimony susceptibility showing CG isolates were pre-adapted to antimony while ISC1 ones were not. This particular feature could be linked to the presence of the H-locus amplification in the CG (Dumetz et al., 2018).

The metabolome being the molecular expression of the parasite's phenotype, its study is extremely relevant to detect important functional differences between organisms. Metabolic differences between ISC1 and CG were found in several functional groups and pathways. First, the most notable changes were found in the lipid metabolism, with 19 GPLs (predominantly GPCs), showing significantly different levels between both groups. Apart from their structural function, GPLs are involved in a wide array of cellular functions including protein and glycoconjugate anchoring, host cell infection, and apoptosis-like processes (Pulido et al., 2017). A second notable metabolic difference concerned the urea cycle. In ISC1 versus CG we detected a higher concentration of citrulline and a lower concentration of argininosuccinate. In organisms with a complete urea cycle, argininosuccinate is then further converted to arginine and fumarate by argininosuccinate lyase (ASL), however, in *Leishmania* this enzyme is missing (Sardar et al., 2016). This difference is of particular interest since both argininosuccinate and arginine deprivation have been linked with reduced thiol content (Sardar et al., 2016; Mandal et al., 2016) and ASS mutants have shown a lower virulence than WT parasites (Lakhal-Naouar et al., 2012). Therefore, the importance of ASS should be further analyzed. Finally, a metabolic change that also came forward in our comparisons between ISC1 and CG strains was the nucleotide salvage pathway. This pathway is essential, since *Leishmania* cannot synthesize the purine ring de novo and is therefore dependent on salvaging these from host purines (Boitz et al., 2012). Our results suggest that ISC1 parasites might be better at salvaging nucleotides from their environment. Altogether, ISC1 and CG parasites appeared to show different levels of activity among different metabolic pathways, all of them susceptible to predict differences in virulence, which should be tested by further work. Notably, our observations were made in stationary phase axenic promastigotes, which contain a high degree of metacyclic, infectious promastigotes (Jamdhade et al., 2015). This is of particular interest, since virulence is of essential importance to this life stage. However, it is certainly possible that more differences between ISC1 and CG might come forward when studying additional parasite life stages in follow-up studies. Ultimately, the rapidly evolving metabolomics technology will most likely allow metabolite quantifications of low amounts of cells in vivo, or even single-cells in the future (Madhubala et al., 2008).

**Fig. 5:**
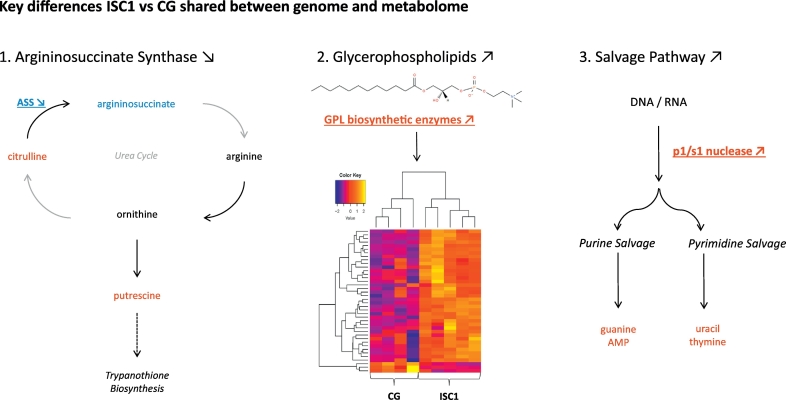
The 3 key differences between ISC1 and CG that are shared between genome and metabolome. 1. Argininosuccinate (ASS) has a lower genomic copy number in ISC1 vs CG. More citrulline and less argininosuccinate were detected in ISC1, suggesting a corresponding downregulation of the ASS protein. Although the urea cycle might be incomplete in *Leishmania*, links have been established with Trypanothione biosynthesis and virulence. 2. Several glycerophospholipid (or precursor) biosynthetic enzymes had higher gene copy numbers in ISC1 which could be linked to increased GPL amounts (See also Fig. 4). 3. More copies of a p1/s1 nuclease gene were found in ISC1, associated with higher concentrations of several salvage pathway metabolites. Genomic copy number information is underlined and in bold. Pathway names are in italics. Arrows in grey represent potentially missing enzymes in the urea cycle of *Leishmania*. Blue = down in ISC1, Red = up in ISC1. (For interpretation of the references to colour in this figure legend, the reader is referred to the web version of this article.)

The parallel untargeted analysis of genome and metabolome of different L. *donovani* strains also allowed us to undertake a first integration of both datasets. A quantitative approach was attempted by directly correlating the pairwise GD-distance and metabolic distances between strains. Although this correlation was found to be significant, they also demonstrate that the genomic distances only explain a limited part of the variation observed at the metabolome level. This is not unexpected, since regulation of gene expression in Trypanosomatids is essentially post-transcriptional. Qualitative analysis of our results showed a few correspondences between genomic and metabolic markers (Fig. 5). Correspondence could concern either an enzyme and the specific metabolites of the reaction it catalyses, or this enzyme and metabolites from the same pathway. The former is best illustrated by the argininosuccinate synthase (ASS) and argininosuccinate, the gene encoding ASS is located on the H-locus and had a 6.7 fold lower expression in ISC1 by the combined effect of somy and local copy number and argininosuccinate is 3.6 times less abundant in the metabolome of ISC1. Two other examples of convergence between genomics and metabolomics concern pathways: genes and metabolites pointing towards a same altered pathway, even if they did not point towards the same product. Firstly, the major changes revealed in the lipid metabolism by metabolomics were mirrored by CNVs. We found 1.55 copies per haploid genome more in ISC1 for diacyl glycerol acyltransferase, which is part of the GPC biosynthesis pathway. Interestingly, we found predominantly higher GPC levels in ISC1. A CNV was also found in a fatty acid elongase (1.65 copies per haploid genome more in ISC1), which is an enzyme responsible for the extension of fatty acids that are later used to build GPLs. Another CNV related to GPL building blocks was found in the glycerol uptake protein. Finally, a CNV was found in 3-ketoacyl-CoA thiolase, which is located in the glycosome and an enzyme of the β-oxidation pathway (breakdown of fatty acids) (Jamdhade et al., 2015). We also detected high impact SNPs in the ISC1 group for two enzymes that are important for lipid signaling: a premature stop codon was in phosphatidylinositol 3&4-kinase and a stop codon was removed from CDP-diacylglycerol-inositol 3-phosphatidyl transferase. Secondly, the different activity of the salvage pathway revealed by metabolomics was also mirrored by genomics. We found indeed that ISC1 parasites have four copies more of the p1/s1 nuclease, which is part of the salvage pathway and responsible for digesting the ssRNA and ssDNA to nucleotides. This was accompanied by an increase of guanine, uracil, thymine and AMP in ISC1. In summary, our data strongly suggest that gene copy number variation may be an important adaptation strategy of the parasite as shown by the gene dosage changes related to metabolic pathways where metabolites were altered (Fig. 5). This implies corresponding changes in protein levels, which is somewhat surprising as transcript levels (driven by gene dosage) are generally assumed to match poorly with protein levels (Madhubala et al., 2008). However, these observations were made in context of differentiation, rather than inter-strains comparisons as we have performed here. Our data suggest that between strains, gene dosage levels might in fact drive protein levels, as was also found in yeast, however, further work is required to define the extent of agreement between variation of genome and proteome.

The evolutionary history and origin of *L*. *donovani* ISC1 are still unclear. In a previous work, genomic data allowed us to estimate the origin of the CG around 1850, hereby fitting with the dates mentioned in the early reports of the first epidemics of “Quinine-resistant malaria” in the ISC, which later on appeared to be VL (Imamura et al., 2016). However, when comparing the number of SNPs between ISC1 and CG and the number of SNPs within CG (45,743 and 2418, respectively, as previously published with a larger sample and an earlier version of the reference genome (3)), we may conclude that divergence time between ISC1 and CG is much earlier than 1850, probably several thousands of years (or more). This suggests that ISC1 and CG represent two post-bottleneck groups of a historically much more polymorphic population endemic in the ISC, although we cannot exclude that one or both were recently introduced from another, currently unsampled VL region. The further fate of ISC1 in the Indian sub-continent remains to be determined by molecular tracking. In the context of the current elimination program, it is possible that new and strong evolutionary bottlenecks may emerge. Among the possible scenarios, the size of the CG population could be strongly reduced, while emerging populations like ISC1 could be favored and spread over the region. Such a scenario is supported by (i) the presence of these variants in regions where the elimination program could be less active, like in the highlands and (ii) the functional differences predicted by the present work.

In summary, we have shown that the ISC1 population is very different in genotype and phenotype from the CG population that drove most recent epidemic of VL in the ISC and that was affected by drug resistance. Genomic and metabolic approaches pointed out major changes in pathways and molecules that are important for the interaction with the host/extracellular environment. Therefore, further work should be performed to deeply characterize ISC1 parasites, especially at functional level. A particular attention is also required to monitor the fate of this emerging population in the ISC, especially in a post-elimination context.

## Funding

This work was supported by the Research Foundation Flanders [11O1614N to B·C]; the Interuniversity Attraction Poles Program of Belgian Science Policy [P7/41 to JC.D.], the InBev Baillet-Latour foundation and the Department of Economy, Science and Innovation in Flanders [ITM-SOFIB, SINGLE to JC.D]. JAC and MS are funded by the Wellcome Trust through their core funding of the Wellcome Trust Sanger Institute [Grant 206194].

## Appendix A Supplementary data

Supplementary Table S1 Image 1

Supplementary Table S2 Image 2

Supplementary Table S3 Image 3

Supplementary Table S4 Image 4

Supplementary Table S5 Image 5
